# Host Determinants of Expression of the *Helicobacter pylori* BabA Adhesin

**DOI:** 10.1038/srep46499

**Published:** 2017-04-18

**Authors:** Mary E. Kable, Lori M. Hansen, Cathy M. Styer, Samuel L. Deck, Olena Rakhimova, Anna Shevtsova, Kathryn A. Eaton, Miriam E. Martin, Pär Gideonsson, Thomas Borén, Jay V. Solnick

**Affiliations:** 1 US Department of Agriculture, Western Human Nutrition Research Center, Davis, CA 95616, USA; 2 Center for Comparative Medicine, University of California, Davis, School of Medicine, Davis, CA 95616, USA; 3 Department of Medical Biochemistry and Biophysics, Umeå University, Umeå SE-90187, Sweden; 4 Department of Microbiology and Immunology, University of Michigan Medical School, Ann Arbor, MI 48109, USA; 5 Departments of Medicine and Microbiology & Immunology, University of California, Davis School of Medicine, Davis, CA 95616, USA

## Abstract

Expression of the *Helicobacter pylori* blood group antigen binding adhesin A (BabA) is more common in strains isolated from patients with peptic ulcer disease or gastric cancer, rather than asymptomatic colonization. Here we used mouse models to examine host determinants that affect *H. pylori* BabA expression. BabA expression was lost by phase variation as frequently in WT mice as in RAG2−/− mice that do not have functional B or T cells, and in MyD88−/−, TLR2−/− and TLR4−/− mice that are defective in toll like receptor signaling. The presence of other bacteria had no effect on BabA expression as shown by infection of germ free mice. Moreover, loss of BabA expression was not dependent on Le^b^ expression or the capacity of BabA to bind Le^b^. Surprisingly, gender was the host determinant most associated with loss of BabA expression, which was maintained to a greater extent in male mice and was associated with greater bacterial load. These results suggest the possibility that loss of BabA expression is not driven by adaptive immunity or toll-like receptor signaling, and that BabA may have other, unrecognized functions in addition to serving as an adhesin that binds Le^b^.

*Helicobacter pylori* infects the gastric mucosa of about 50% of the world’s population^1^. The majority of those infected have only asymptomatic gastritis, but about 10% develop peptic ulcer and 1–3% develop gastric cancer^1^,^2^,^3^, which is the third most common cause of cancer death worldwide (~1 million cases per year). Given the large number of infected individuals, increasing development of antibiotic resistance^4^,^5^, and accumulating evidence that in some people *H. pylori* may be beneficial^6^,^7^,^8^,^9^, treatment of all infected individuals may not be warranted. Therefore, it is important to determine the elements of the host-*H. pylori* interaction that influence whether an individual will develop clinical disease or asymptomatic infection. One risk factor associated with more severe disease outcomes is the virulence factor, blood group antigen binding adhesin (BabA), which belongs to a family of *H. pylori* outer membrane proteins^10^ that also includes LabA^11^, SabA^12^, and the recently characterized HopQ^13^,^14^. BabA is a well-characterized adhesin^15^,^16^,^17^,^18^ that binds to ABO blood group antigens, fucosylated carbohydrates expressed on the gastric epithelium and the protective mucus layer. BabA exhibits highest affinity for Lewis b (Le^b^)^19^, owing to a polymorphic, three-pronged carbohydrate binding domain identified recently by X-ray structural analysis^20^,^21^. Epidemiologic studies of an association of BabA with disease^22^,^23^ are supported by *in vitro* evidence that BabA-mediated attachment to host gastric epithelium facilitates translocation of the CagA oncoprotein into host cells^24^. Translocation occurs via the type IV secretion system encoded on the cytotoxin associated gene pathogenicity island (*cag*PAI), itself a well-recognized risk factor for disease^25^,^26^,^27^,^28^,^29^.

BabA mediated attachment and development of disease are influenced by host expression of Lewis antigens, which is determined by a number of factors, including ABO blood type and secretor status^30^,^31^,^32^,^33^. The risk of ulcer is increased in individuals with blood group O, and in non-secretor individuals who do not express Le^b^ and ABO antigens on gastric epithelial cells or on mucins^30^,^31^,^32^,^33^,^34^. Thus, disease outcome is related to both bacterial expression of the BabA adhesin and to ABO glycosylation on gastric epithelial cells and gastric mucins.

Previous studies have shown that *H. pylori* expression of BabA is lost during the first 2–12 weeks of infection using animal models such as rhesus macaques, gerbils, and mice^35^,^36^,^37^. Loss of BabA expression has been observed to occur by two mechanisms. In rhesus macaques, recombination between the *babA* gene and its *babB* paralog can result in duplication of all or part of the *babB* gene into the *babA* locus, resulting in loss of BabA expression and reciprocal overexpression of BabB. Alternatively, in mice and in macaques, slipped strand mispairing of a CT repeat region in the 5′ portion of the *babA* open reading frame (ORF) can lead to a frame shift, resulting in an early stop codon within the *babA* ORF and loss of BabA protein expression. That this phenomenon occurs across several animal models, and that loss of BabA expression can also be seen in human clinical isolates^38^, suggests that modulation of BabA expression is an important component of the *H. pylori*-host relationship.

Loss of BabA expression might occur as a bacterial mechanism of persistence to adapt to changing levels of inflammation, glycosylation patterns, or a combination of both. Here we used knockout and transgenic mouse models to determine the host factors that affect BabA expression. Surprisingly, the results suggest that adaptive immune responses and toll-like receptor signaling play little if any role in loss of BabA expression, and that the capacity to bind Le^b^ is not required, but that gender- specific physiological differences may be important.

## Methods

### Bacterial strains and growth conditions

A complete list of bacterial strains used in this study is provided in Table 1. *H. pylori* cultures were maintained on Brucella agar (Becton, Dickinson and Company, Sparks, MD) supplemented with 5% heat inactivated newborn calf serum (Gibco, Grand Island, NY) and antibiotics (Sigma-Aldrich, Inc., St. Louis, MO), either TVPA (5 μg/mL trimethoprim, 10 μg/mL vancomycin, 2.5 units/mL polymyxin B and 2.5 μg/mL amphotericin B) for laboratory adapted cultures or ABPNV (100 μg/mL vancomycin, 3.3 μg/mL polymixin B, 200 μg/mL bacitracin, 10.7 μg/mL nalidixic acid and 10 μg/mL amphotericin B) for primary cultures from infected mice. All *H. pylori* cultures were maintained at 37 °C in a CO_2_ incubator or in an Anoxomat^TM^ jar (Advanced Instruments, Inc., Norwood, MA) adjusted to contain 5% oxygen, 7.6% carbon dioxide, and 7.6% hydrogen^39^.

### Site directed mutagenesis

Isogenic mutants of *H. pylori* J166 were engineered with 7 (no ORF) or 8 (ORF) CT repeats in the 5′ coding region of *babA* using a contraselection approach modified from that previously described^36^. Briefly, 244 base pairs upstream of the *babA* translational start site and 39 base pairs of 5′ *babA* gene sequence were replaced with a CAT_rpsL cassette in a streptomycin resistant strain of *H. pylori* J166, using the plasmid pBabA as previously described^39^. The CAT_rpsL cassette was amplified using primers RpsLF and CamR (Table 2)^36^. The pBabA plasmid was constructed by ligating CAT_rpsL sequence between two PCR amplicons. The first was a 1211 bp amplicon containing *hypD* and intergenic sequence 244 bp upstream of *babA* in *H. pylori* J166 (primers HypDF and BabApromR, Table 2), and the second amplicon contained 996 bp of *babA* starting 39 bp downstream of the *babA* start site (primers BabAF and BabAR, Table 2). The resulting *babA* knockout strain was then transformed using genomic DNA from primary mouse output strains containing either 7 or 8 CT repeats in the 5′ coding region of *babA*. Transformants containing the desired number of CT repeats were selected by plating on streptomycin followed by replica plating to confirm loss of chloramphenicol resistance. The same approach was used to generate 8 (ORF) or 9 (no ORF) CT isogenic variants of J166 BabACL2, a site directed mutant of J166 in which Cys to Ala replacements at residues 189 and 197 result in a BabA protein that is expressed but cannot bind Le^b^ 20. All mutants were sequenced using the method of Sanger to confirm the correct CT repeat structure.

### Mouse strains, housing and breeding

The local Institutional Animal Care and Use Committee approved all mouse experiments performed at Should read, University of California, Davis and the University of Michigan. Experiments performed at Umeå University, Umeå, Sweden were approved by Umeå Ethical Committee on Animal Research. All methods involving mice were performed according to the locally approved guidelines. Mice (Supplementary Table S1) were housed in sterilized, ventilated microisolator cages and given autoclaved water and irradiated food *ad libitum* as previously described^36^. FVB/N mice heterozygous for human α-1,3/4-fucosyltransferase gene (Le^b^ transgenic mice)^40^ were bred to FVB/N WT mice and genotyped using tail snips obtained at weaning. DNA was extracted from tail snips by digestion in lysis buffer (50 mM KCl, 10 mM Tris, pH 8.5; 2 mM EDTA, Sigma-Aldrich, St. Louis, MO; 0.45% NP-40, Roche Diagnostics, Indianapolis, IN; 0.45% Tween-20, Bio-Rad, USA; and 1 mg/mL proteinase K, Roche Diagnostics, Indianapolis, IN) for 2 hr or overnight. Undigested fragments were removed by centrifugation (8,500 g for 10 min) and proteinase K was heat inactivated at 98 °C for 10 min. The resulting digest was used as template for PCR detection of human α-1,3/4-fucosyltransferase gene using primers hGh-F and hGh-R (Table 2), which were previously described^41^. Germ free C57BL/6 mice were raised and housed in soft-sided bubble isolators at the germ free mouse facility at the University of Michigan. Germ-free status was verified by aerobic and anaerobic cultures at least weekly, and all mice remained free of bacteria (other than the inoculated *H. pylori* strain) throughout the experiment.

### Experimental *H. pylori* challenge and sample collection

*H. pylori* harvested from agar plates grown overnight (18–24 hr) was used to inoculate liquid cultures to an optical density A_600_ (OD) of 0.05 to 0.1. Liquid cultures were grown in Brucella broth (Becton, Dickinson and Company, Sparks, MD) supplemented with 5% NCS and TVPA shaking at 60–100 rpm overnight (18–24 hr) to an OD of 0.3 to 0.7. Bacteria from overnight liquid cultures were centrifuged (7,000 g for 10 min) and suspended in Brucella broth at approximately 10^10^ CFU/mL. For competition experiments where the inoculum contained equal parts of BabA expressing and deficient *H. pylori*, liquid cultures were grown to matching OD values prior to centrifugation and resuspension. In a subset of experiments, dilutions of the inoculum mixture were plated and 16 colonies were selected and sequenced at the 5′end of the *babA* locus to empirically determine the ratio of BabA expressing bacteria in the inoculum. On average the inoculum contained 45% BabA expressing bacteria with a standard deviation of 11%. This variability reflects both variation in the growth and survival of the cultures and technical variation in the detection of BabA expression in the output colonies. Mice were challenged at 10 to 14 weeks of age with 250 μL of suspension by oral gavage with a 20 gauge, 38mm animal feeding needle (Fisher Scientific)^36^. Mice were euthanized by intraperitoneal injection of 5 mg pentobarbitol-Na and 0.6 mg phenytoin-Na (Beuthanasia-D) and stomachs were removed and dissected into 2 to 4 longitudinal sections^36^,^42^. One quarter of the stomach tissue was placed in 10% phosphate buffered formalin (Fisher Scientific) for histological analysis and one half to one quarter was weighed and homogenized in Brucella broth for *H. pylori* culture and calculation *of H. pylori* colony forming units per gram of stomach tissue (CFU/g). The comparison between male and female mice was performed twice, as indicated in the figure legends and supplementary files. The remainder of the experiments was conducted exclusively in female mice.

### Detection of BabA expression

In *H. pylori* J166, 8 CT repeats at the 5′ end of *babA* yield an ORF, with expression of BabA and attachment to Le^b^ 36; 7 or 9 CT repeats does not because it produces a stop codon at position 49 or 79, respectively. Therefore, the proportion of BabA-expressing *H. pylori* in each mouse was determined by sequencing the CT repeat region of *babA*, which was selectively confirmed by RIA analysis using methods previously described^36^,^43^. Briefly, for CT analysis, a DNA fragment was sequenced from multiple individual colonies from each mouse after PCR amplification with primers BabAF14 and BabARJC2 (Table 2), which yields a 1046 bp product containing the CT repeats. On average 8 colonies were collected per mouse and analyzed in this way (minimum of 3 and maximum of 20). The percentage of the total colonies collected per mouse with a *babA* ORF is displayed as a single point in each figure describing BabA expression in mice. For RIA analysis, total cultured dilutions from mouse stomach homogenate (sweeps) were collected and 1 mL of an OD_600_ = 0.1 suspension of the mixture was incubated with a cocktail of ^125^I radiolabeled Le^b^ conjugated to human serum albumin (Le^b^-HSA). The ratio of bound Le^b^-HSA (radioactivity measured in the bacterial pellet) to free Le^b^-HSA (radioactivity measured in the supernatant) was used to estimate BabA expression of the *H. pylori* community^43^.

### *In vitro* analysis of BabA attachment to Le^b^

*H. pylori* expression of functional BabA protein was assayed by *in vitro* attachment to Le^b^ using an ELISA as previously described^22^,^35^,^43^. Briefly, digoxigenin (Roche Applied Biosciences) labeled *H. pylori* cells were applied to Le^b^-HSA coated wells in a 96 well polystyrene plate. Unbound bacteria were removed by washing and bound bacteria were detected with anti-digoxigenin Fab fragments conjugated to horseradish peroxidase (POD) (Roche Applied Biosciences) followed by incubation with 2,2′-azino-di(3-ethyl-benzthiazoline-6-sulfonate) (ABTS). Color change was measured by subtraction of absorbance at 490 nm from 405 nm. Attachment ratio values were reported as an average of readings from two Le^b^ positive wells divided by two Le^b^ negative wells.

### Statistical Analysis

Unless otherwise indicated, Mann-Whitney U, Fisher’s exact test or analysis of variance was performed using Graphpad Prism Software. Bacterial colonization (CFU/gram) was log transformed prior to analysis. The proportion of BabA expressing colonies was logit transformed using R software^44^ and 0 values were remapped to a proportion of 0.025 prior to analysis of variance. If no bacteria were isolated from a given mouse, the colonization level was shown at the limit of detection. *P* < 0.05 was considered statistically significant.

## Results

### Adaptive immunity does not affect *H. pylori* BabA expression

**Figure 1:**
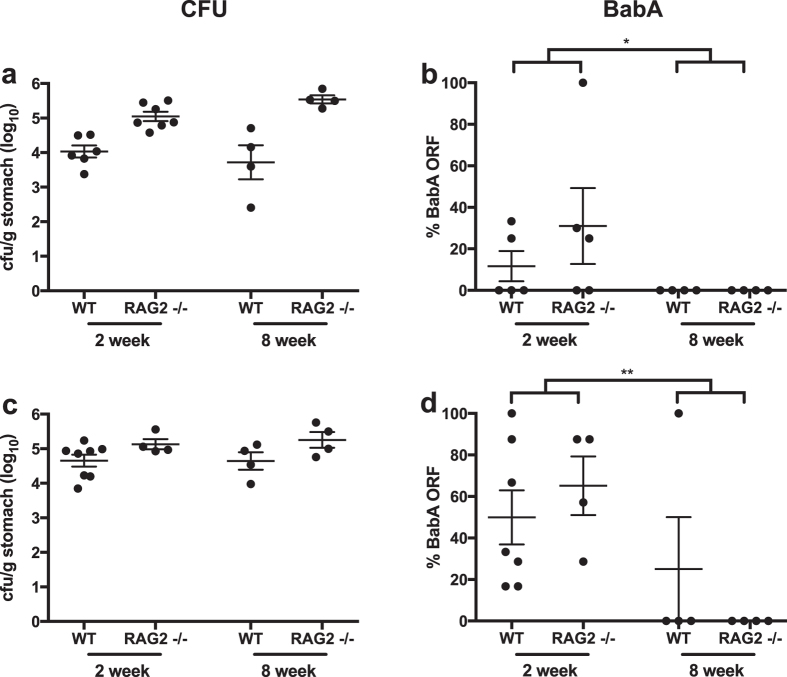
Loss of BabA expression in mice is not affected by B or T cell response. *H. pylori* colonization was significantly greater in female RAG2−/− compared to WT mice at 2 and 8 weeks PI when the results were pooled over time, *P* < 0.0001 (**a**), but loss of the *babA* ORF was unaffected (**b**). Each point represents the percentage of colonies (average of 8 colonies total) from a single mouse determined by sequencing to express a full length *babA* ORF. Similar results were obtained when the experiment was repeated (**c**,**d**), although the difference in colonization level was less significant, *P* < 0.05 (**c**). Data were analyzed by two-way ANOVA of log (**a**,**c**) or logit (**b**,**d**) transformed values. *P < 0.05, **P < 0.01.

Loss of *H. pylori* BabA expression by phase variation might result from adaptive immune pressure directed against BabA. When loss of BabA expression occurs by a gene conversion event in which *babB* is duplicated into the *babA* locus^35^,^36^, this might represent a form of antigenic variation. Both occur commonly in bacteria and other pathogens to avoid adaptive immunity^45^,^46^,^47^,^48^. To test this hypothesis, we measured BabA expression in output strains after inoculation of *H. pylori* J166 into wild type (WT) and RAG2−/− mice, which do not develop mature B or T lymphocytes^49^. *H. pylori* colonized RAG2−/− mice at a significantly higher level than WT (Fig. 1a), supporting previous evidence that adaptive immunity, including mature T lymphocytes^50^,^51^, is important for control of *H. pylori* infection. To examine BabA expression, 3 to 6 *H. pylori* colonies were isolated from the stomachs of RAG2−/− and WT mice (N = 4–7) at 2 and 8 weeks post infection (PI). Since we previously showed that loss of BabA expression in mice occurs only by phase variation and not by gene conversion^36^, we determined BabA expression by DNA sequence analysis of the 5′ region that contains the CT dinucleotide repeats, where 8 repeats corresponds to a *babA* open reading frame (ORF) and attachment to Le^b^ 36. Although there was considerable variability among individual mice 2 weeks PI, by 8 weeks PI BabA expression was lost in 8 of 8 mice (Fig. 1b). No differences were found between RAG2−/− and WT mice. The experiment was repeated with similar results (Fig. 1c,d). These data indicate that although the adaptive immune response controls *H. pylori* infection, it does not play a role in selection against BabA expressing bacteria in mice.

### *H. pylori* BabA expression is lost in both conventional and germ free mice

**Figure 2:**
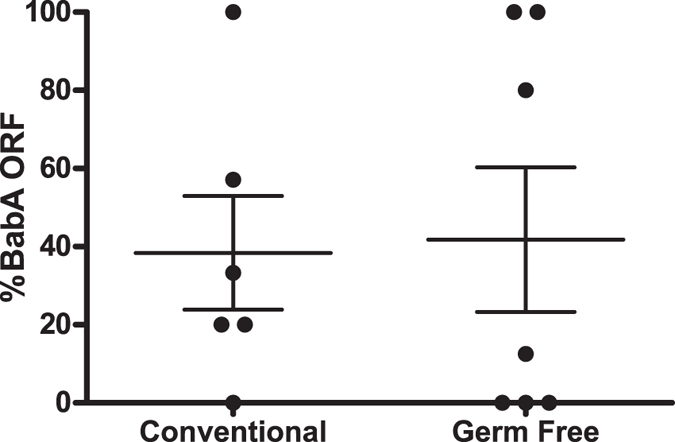
*H. pylori* loss of BabA expression occurs equally in germ free and conventionally raised mice. Percent BabA expressing *H. pylori* is shown for conventional and germ free female C57BL/6 mice inoculated with *H. pylori* J166 and sacrificed 3 weeks PI. No significant differences were found between the experimental groups.

It is now recognized that *H. pylori* infection occurs in the context of a gastric microbial community, which is less complex than that in the gut, but is probably autochthonous^52^,^53^,^54^,^55^ and may affect the outcome of infection. For example, in INS-GAS mice, which overexpress gastrin under the insulin promoter^56^, *H. pylori* infection induces more severe gastrointestinal intraepithelial neoplasia when animals have conventional microbiota compared to germ free counterparts^57^. Additionally, differences in intestinal microbiota composition in mice from different vendors have been shown to impact the immune response to pathogens^58^. Since adaptive immunity did not select for loss of BabA expression, and there was substantial variability among individual mice during the first two weeks of infection, we considered the possibility that the gastric microbial community could affect BabA expression. To determine whether microbiota could influence *H. pylori* expression of BabA, we infected germ free and conventionally raised C57BL/6 mice with *H. pylori* J166, and examined BabA expression in output colonies from mice sacrificed 3 weeks PI. Similar to the results 2 weeks PI (Fig. 1), there was marked variability, but no statistical differences in loss of BabA expression between conventional and germ free C57BL/6 mice (Fig. 2). These results indicate that the microbiota does not affect *H. pylori* expression of BabA, nor influence the variability among individual mice.

### The *H. pylori* J166 inoculum is heterogeneous at the BabA locus

The marked variability in BabA expression among output colonies (Figs 1 and 2) might result from stochastic or bottleneck effects if the inoculum contains a significant population of phase variants with no *babA* ORF. This possibility was supported by the finding that sequence of 5–6 individual colonies from 3 instances of liquid culture grown from the same *H. pylori* J166 stock contained variable percentages of BabA expressing clones, ranging from 67 to 100%. We therefore re-isolated a J166 single colony (designated J166_SC_) with a *babA* ORF (8 CT repeats) and compared the outcome of infection with J166sc to that with the original J166 inoculum containing a mixture of phase variants. After infection for between 2 and 8 weeks, loss of BabA expression was observed in 6 of 11 (55%) mice infected with the original stock of J166, but only 1 of 12 (8%) mice infected with J166sc (*P* < 0.05, Fisher’s exact test). Since J166_SC_ might differ from J166 in other ways, in addition to the number of CT repeats in *babA*, we used contraselection to generate isogenic clones of J166_SC_ that had either 8 CT repeats (J166_8CT_), encoding a *babA* ORF, or 7 CT repeats (J166_7CT_), which was out of frame and led to loss of BabA expression. J166_8CT_ and J166_7CT_ showed similar growth rates in liquid culture (Supplementary Figure S1), but, as expected, only J166_8CT_ attached to Le^b^ (Supplementary Figure S2). For all subsequent experiments, *H. pylori* inoculation was performed as a competition experiment in which mice were infected with an equal mixture of J166_8CT_ and J166_7CT_ (designated J166_8CT:7CT_).

### BabA expression decreases within 24 hours during acute infection

**Figure 3:**
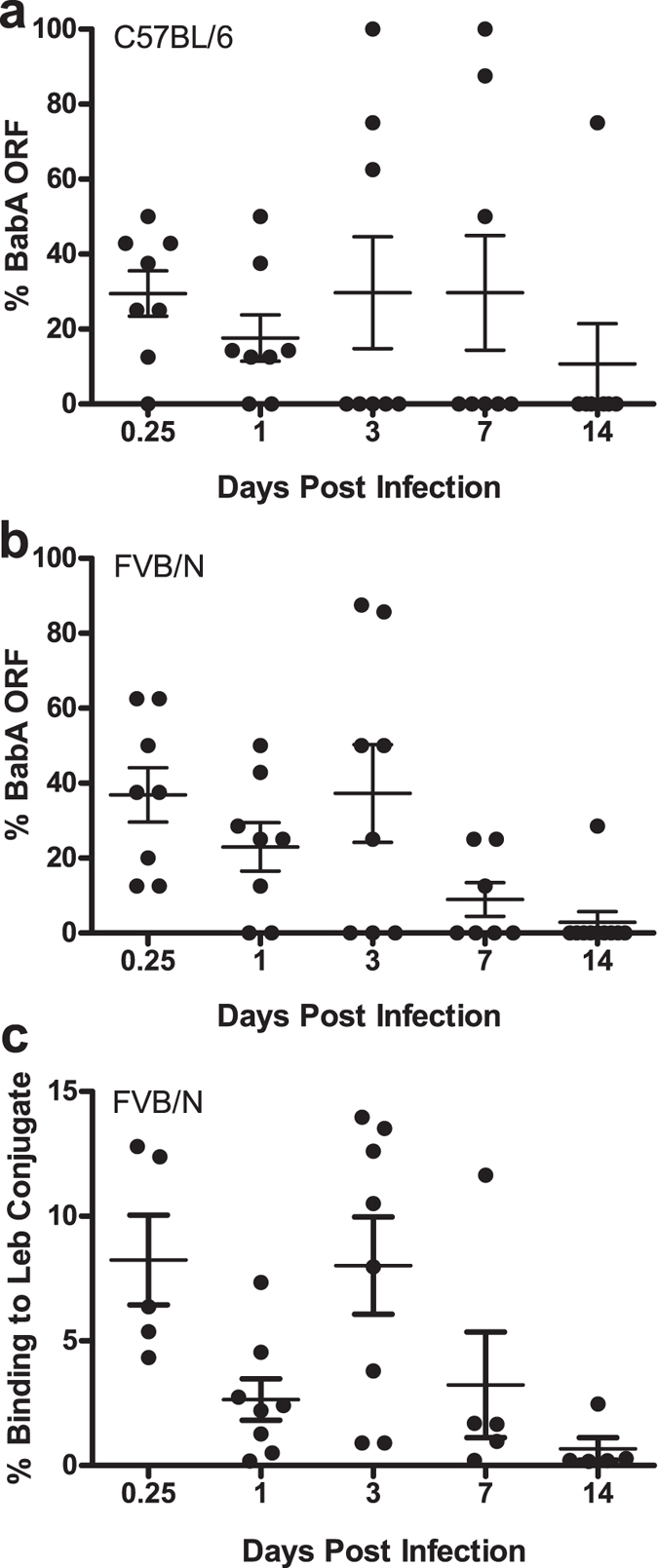
*H. pylori* loss of BabA expression occurs early during infection. Percentage of BabA expressing *H pylori* detected by CT repeat analysis in female C57BL/6 (**a**) and female FVB/N (**b**) mice inoculated with J166_8CT:7CT_ and sacrificed from 6 hrs to 14 days PI. (**c**) *H. pylori* populations cultured from the stomachs of FVB/N mice shown in B, analyzed for binding to Le^b^-HSA conjugate by RIA analysis.

Previous experiments indicated that loss of BabA expression can occur within the first two weeks of *H. pylori* infection (Fig. 1). We next examined this more precisely by inoculating WT C57BL/6 mice with J166_8CT:7CT_ and examining BabA expression in output colonies recovered from mice sacrificed between 6 hrs and 14 days PI. Loss of BabA expression was detected as early as 6 hrs PI, was dominant by 3 days PI, and nearly uniform at 2 weeks PI (Fig. 3a). To confirm that this was not specific to a particular mouse strain, we performed the same experiment in WT FVB/N mice. Loss of BabA expression was similar in FVB/N mice (Fig. 3b) to what we previously observed in C57BL/6 mice (Fig. 3a), with colonies from 10 of 11 mice (91%) showing no BabA expression by 2 weeks PI. To confirm that sequencing the *babA* CT repeat region in a limited number of output colonies is representative, we performed RIA analysis, which detects Le^b^ binding in the total *H. pylori* population cultured from the stomach. Le^b^ binding analyzed by RIA correlated closely with CT analysis (Pearson R^2^ = 0.57, *P* < 0.0001), and demonstrated a similar loss in BabA expression over time (Fig. 3c). Together, these data suggest that there is a strong selection against BabA expression in mice that occurs within hours to a few days after inoculation.

### Role of toll like receptor (TLR) signaling in BabA expression

**Figure 4:**
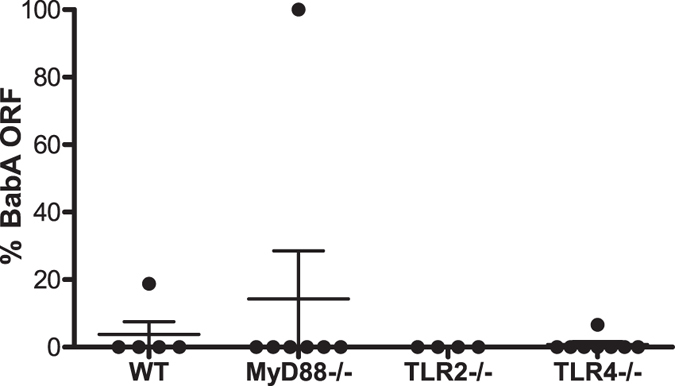
TLR signaling does not influence *H. pylori* BabA expression. Percentage of BabA expressing *H. pylori* detected in female C57BL/6 WT, MyD88−/−, TLR2−/− and TLR4−/− mice inoculated with J166_8CT:7CT_ and sacrificed 2 weeks PI. No significant differences were found between any of the experimental groups.

Early selection, hours to days post infection, against BabA expression is consistent with the absence of a role for adaptive immunity in selecting against BabA expression (Fig. 1), and suggests that innate immunity might play a role. Attachment to host epithelial cells has been shown to induce expression of IL-8^24^, suggesting that attachment increases host innate inflammatory responses and perhaps selects against strains expressing BabA. Moreover, we previously found that BabA expression was retained to a greater extent in C3H/HeJ mice^36^, which express a defective toll like receptor 4 (TLR4)^59^. Together, these data suggest the possibility that TLR signaling could be involved in driving loss of BabA expression. To examine this, BabA expression was analyzed 2 weeks PI in C57BL/6 WT mice and compared to mice with a homozygous deletion of MyD88 (MyD88−/−), toll like receptor 2 (TLR2−/−), or TLR4−/−. By 2 weeks PI, no BabA expressing bacteria were detected in the large majority of mice from each experimental group (Fig. 4), indicating that loss of BabA expression is under strong negative selection even in the absence of TLR signaling.

### *H. pylori* BabA expression is greater in male mice

**Figure 5:**
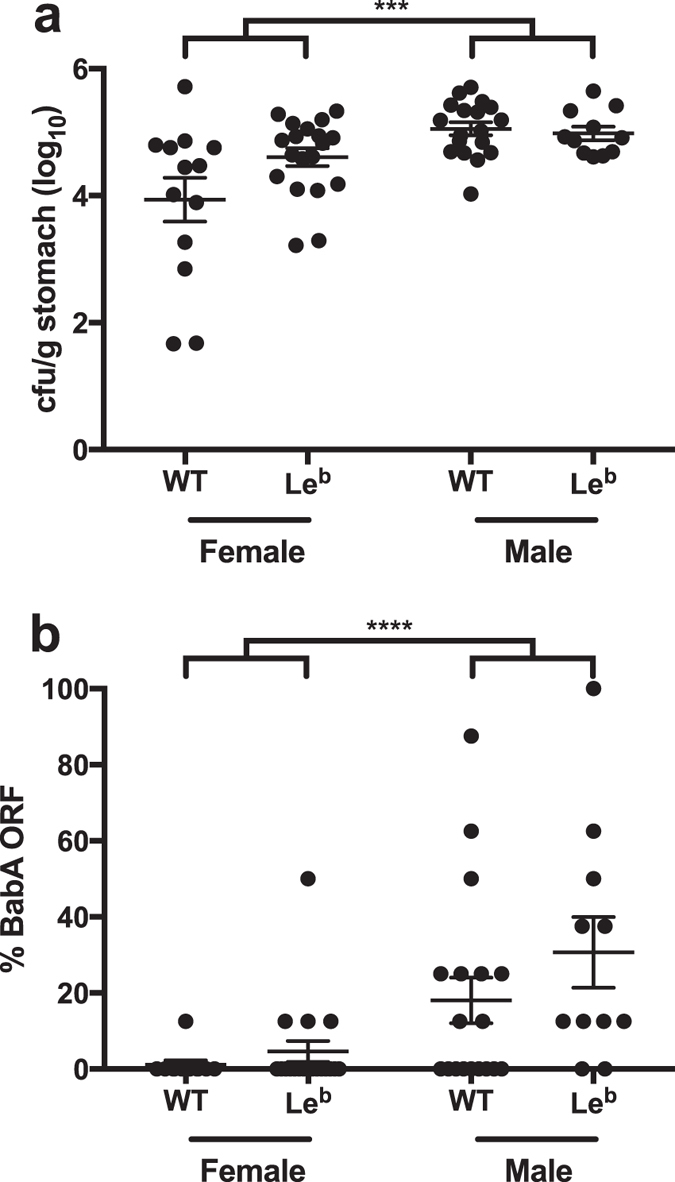
*H. pylori* colonization and BabA expression are greater in male than female FVB/N mice. Male and female Le^b^ transgenic FVB/N mice and their WT littermates were inoculated with J166_8CT:7CT_ and sacrificed 2 weeks PI. *H. pylori* colonization density (**a**) and expression of BabA (**b**) were greater in male than in female mice. ***P < 0.001, ****P < 0.0001 (Two-way ANOVA of logit (**a**) or log (**b**) transformed values).

Glycosylation levels and patterns are altered early during *H. pylori* infection, with loss of fucosylation and increase in sialylation due to inflammation-induced changes in glycosyltransferases^12^,^36^,^60^,^61^,^62^. Specific glycans can affect not only adherence, but also *H. pylori* growth and gene expression^63^. We therefore compared BabA expression in WT mice, which do not express the Le^b^ antigen, to transgenic mice that express Le^b^ in the gastric mucosa^40^. Male and female Le^b^ transgenic mice and their wild type littermates were infected with J166_8CT:7CT_ and sacrificed 2 weeks PI. *H. pylori* colonization levels were greater in males (Fig. 5a, *P* < 0.001), but there was no difference between WT and Le^b^ mice. BabA expression was also similar in WT and Le^b^ mice, but again there was a significant effect of gender (Fig. 5b, *P* < 0.0001). Female mice were predominantly colonized by *H. pylori* lacking BabA expression, similar to what we observed previously (Figs 3 and 4), while BabA expression was greater in male mice (Fig. 5b). Expression of BabA in *H. pylori* isolated from male Le^b^ mice was significantly greater than that from either female Le^b^ mice or female WT mice (Tukey’s post test, *P* = 0.002 and *P* = 0.001, respectively). Increased colonization and a small increase in Le^b^ binding were also observed in male Le^b^ relative to female Le^b^ mice 4 weeks PI using *H. pylori* strain J99 (Supplementary Figure S3), indicating that this phenomenon is robust across *H. pylori* strains that express functional BabA.

### Loss of *H. pylori* BabA expression is not dependent on BabA attachment to Le^b^

**Figure 6:**
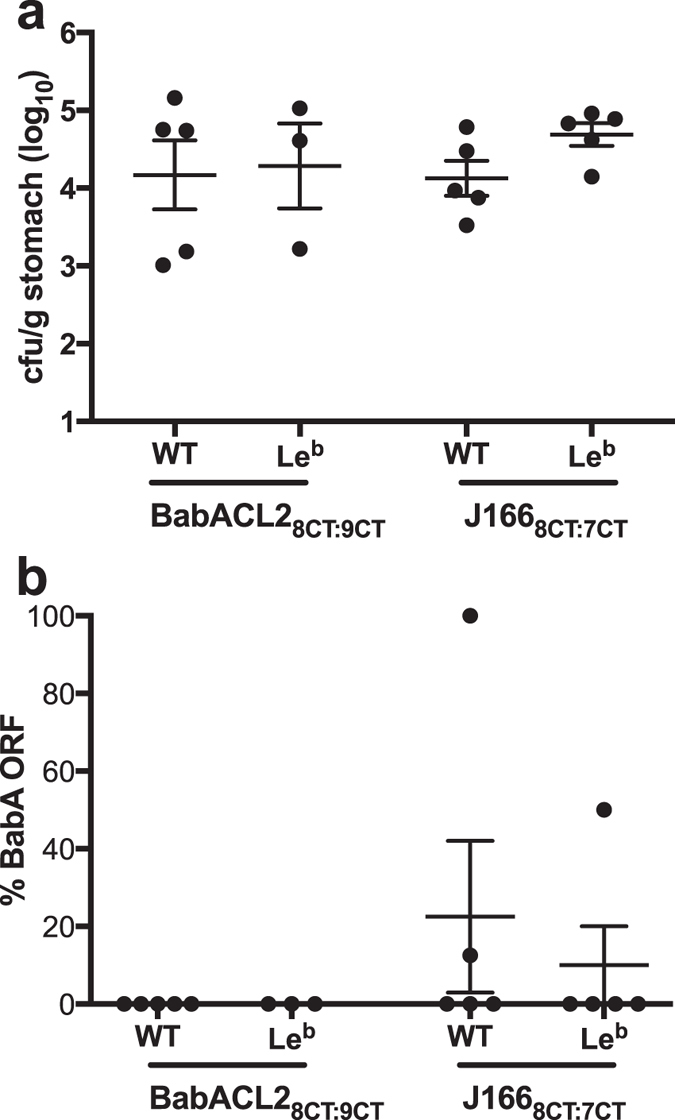
Loss of BabA expression in mice occurs even in the absence of capacity to bind Le^b^. Female Le^b^ transgenic FVB/N mice and their WT littermates were inoculated with BabACL2_8CT:9CT_ or J166_8CT:7CT_ and sacrificed 2 weeks PI. Colonization density (**a**) and selection for loss of BabA expression (**b**) were similar in WT and Leb mice inoculated with J166 and BabACL2, which expresses BabA but does not attach to Leb. No significant differences were found between any of the experimental groups.

Since loss of BabA expression in mice is not significantly affected by expression of Le^b^, we hypothesized that it might also be unaffected by the capacity of BabA to bind Le^b^. We recently demonstrated that BabA residues Cys189 and Cys197 form a redox-sensitive disulfide-clasped loop designated CL2, which is essential to bind the Le^b^ α-1-2-linked fucose residue^20^. Cys to Ala replacement at BabA residues 189 and 197 in *H. pylori* J166 (designated BabACL2) was sufficient to eliminate all Le^b^ binding activity, though the protein was expressed on the cell surface at levels similar to WT^20^. To examine the effect of Le^b^ binding on BabA expression in mice, we used contraselection to generate isogenic strains of BabACL2 with either 8 (J166 BabACL2_8CT_) or 9 (BabACL2_9CT_) CT repeats. The BabA expression status predicted by the CT repeat number for each strain was confirmed by western blot (Supplementary Figure S4). The elimination of Le^b^ binding activity in all strains was confirmed by ELISA (Supplementary Figure S4). These strains were combined in equal proportions (BabACL2_8CT:9CT_) and used to infect female Le^b^ transgenic mice and their WT littermates, which were sacrificed 2 weeks PI. For comparison, WT and Le^b^ mice were also infected with J166_8CT:7CT_. There were no differences in colonization levels between BabACL2_8CT:9CT_ compared to J166_8CT:7CT_ (Fig. 6a) in WT or in Le^b^ mice. Strikingly, there was strong selection for loss of BabA expression, even in the absence of the capacity to bind Le^b^ (Fig. 6b).

## Discussion

We previously found that expression of BabA was lost either by phase variation or by gene conversion within the first 2–12 weeks during experimental *H. pylori* infection of non-human primates, mice, and gerbils^35^,^36^. Similar observations have been made by others^37^,^64^. Clinical isolates of *H. pylori* also show remarkable diversity at the *babA* locus, which may encode a “specialist” adhesin that binds only blood group O/Le^b^, or a “generalist” that also binds blood group A/ALe^b^ and B/BLe^b^ 19,20. In other cases, BabA may be expressed but not bind any known blood group antigen, may be present as a pseudogene, or even be absent from the genome altogether^36^. We recently suggested that in order to persist in the human stomach, *H. pylori* must face what we called an “attachment dilemma”, in which the benefits of adherence to the gastric epithelium such as escape from luminal acid and nutrient acquisition must be balanced with the costs, particularly interaction with the host immune response^65^. This is likely a dynamic process where BabA expression can be lost but also regained, either by phase variation, or perhaps by reintroduction of a copy of *babA* that has been archived in a small proportion in the population^66^. The diversity and dynamic nature of host glycosylation further adds to the complexity. With this perspective, we hypothesized that modulation of BabA expression and attachment to Le^b^ would be driven by the host immune response and glycan expression. Here we set out to test this hypothesis.

Surprisingly, our results suggest that neither adaptive immunity, toll like receptor mediated host immune responses, Le^b^ expression, nor even the capacity of BabA to bind Le^b^ affected loss of BabA expression in mouse models. BabA expression was lost by phase variation equally in WT mice as in RAG2−/− mice that do not have functional B or T cells, and in MyD88−/− mice that cannot signal via all TLRs except TLR 3. Initial experiments suggested that loss of BabA expression required up to 8 weeks of colonization (Fig. 1), which might suggest involvement of host immunity, but competition experiments demonstrated selection for loss of BabA expression as early as one day PI (Fig. 3). Although we have not specifically examined the role of innate immune cells such as polymorphonuclear leukocytes (PMNs) and macrophages, our results suggest that adaptive immunity and TLR mediated immune responses do not select for loss of BabA expression. Moreover, loss of BabA expression is not dependent on Le^b^ expression or the capacity of BabA to bind Le^b^, which suggests the possibility that BabA may have other, unrecognized functions.

Instead, we found that gender is the host determinant most associated with loss of BabA expression, which was maintained to a greater extent in male mice than in females, and was also associated with greater bacterial load (Fig. 5 and Supplementary Figure S3). These data highlight the importance of conducting host-pathogen interaction studies in both genders when using a mouse model, which is a common experimental design problem that has gained increased interest in recent years^67^. Male gender is a well-known risk factor for *H. pylori*-associated disease, including gastric adenocarcinoma^68^ and peptic ulcer, though the male predominance in ulcers appears to be declining as its prevalence decreases^69^. Animal models also provide support for male predominance of gastric cancer^70^. Although less studied, there is also epidemiologic evidence that male gender is a risk factor for *H. pylori* infection^71^, and that, as we observed, *H. pylori* bacterial load in mouse models is higher in males than in females^72^,^73^.

The mechanistic link between host gender and *H. pylori* expression of BabA remains to be determined. There are numerous differences in gastric physiology between males and females^74^, including pH, transit time, and enzyme expression, though it is unclear how these or other differences might affect relative fitness of *H. pylori* BabA expression. Similarly, gender dependent differences in glycosylation patterns have been described in humans^75^,^76^,^77^, suggesting the possibility that differences in mucosal glycan expression between male and female mice might participate in selection against BabA expression. However, since capacity to bind Le^b^ is not required for loss of BabA expression, the effect of gender differences in glycosylation may involve as yet uncharacterized lectin functions of BabA.

## Additional Information

**How to cite this article:** Kable, M. E. *et al*. Host Determinants of Expression of the *Helicobacter pylori* BabA Adhesin. *Sci. Rep.* **7**, 46499; doi: 10.1038/srep46499 (2017).

**Publisher's note:** Springer Nature remains neutral with regard to jurisdictional claims in published maps and institutional affiliations.

## Supplementary Material

Supplementary Files

## Figures and Tables

**Table 1:** Bacterial strains.

Strain	Description	Antibiotic Resistance^a^	Source (Reference)
J166	Wild Type		35
J99	Wild Type		78
26695	Wild Type		36,78
J166Δ*babA*upstream	J166 with the upstream portion of *babA* replaced by CAT_rpsL (transformed with pBabA)	Cm	This study
J166_sc_	Single colony isolate of J166	Str	This study
J166_7CT_	J166 engineered with 7 CT repeats in the 5′ end of *babA*	Str	This study
J166_8CT_	J166 engineered with 8 CT repeats in the 5′ end of *babA*	Str	This study
J166Δ*babA*	J166 with *babA* replaced by CAT_rpsL	Cm	20
J166 BabACL2	J166 with Cys 189 and 197 in BabA replaced with Ala	Str	20
J166 BabACL2_8CT_	J166BabACL2 engineered to have 8 CT repeats in the 5′ end of *babA*, non-binding BabA is expressed	Str	This study
J166 BabACL2_9CT_	J166BabACL2 engineered to have 9 CT repeats in the 5′ end of *babA*, BabA is not expressed	Str	This study

^a^Cm, chloramphenicol; Str, streptomycin.

**Table 2:** Primer sequences.

Primer	Restriction Site	Sequence (5′-3′)^a^
Construction of pBabA		
RpsLF	SacI	AAC GAGCTC GAT GCT TTA TAA CTA TGG ATT AAA CAC
CamR	BamHI	AAC GGATCC TTA TCA GTG CGA CAA ACT GGG AT
HypDF	NotI	AAC GCGGCCGC AGC CAC AAA ACC TCT AAA GA
BabApromR	SacI	AAC GAGCTC GGG GTA TTT TGA AAT AAC TCT C
BabAF	BamHI	AAC GGATCC TTG CTC CAC GCT GAA GAC
BabAR	XhoI	AAC CTCGAG GAC GCT CGT TTG ATT GAC CA
Le^b^ genotyping		
hGh-F		AGC TGG CCT TTG ACA CCT ACC AGG
hGh-R		TCT GTT GTG TTT CCT CCC TGT TGG
CT repeat length determination		
BabAF14		GCA TCA AGC AAG CGA TAA CTT TAC TAA
BabARJC2		TTT GCC GTC TAT GGT TTG G

^a^Restriction sites are underlined.
