# Supplementary material for: Host Determinants of Expression of the Helicobacter pylori BabA Adhesin
Source: Sci Rep. 2017 Apr 18;7:46499. doi: 10.1038/srep46499 (PMC5394467; doi:10.1038/srep46499)
Supplement: Supplementary Files [file srep46499-s1.pdf]

Host Determinants of Expression of the *Helicobacter pylori* BabA Adhesin

Mary E. Kable, Lori M. Hansen, Cathy M. Styer, Samuel L. Deck, Olena Rakhimova, Anna Shevtsova, Kathryn A. Eaton, Miriam E. Martin, Pär Gideonsson, Thomas Borén, and Jay V. Solnick

Supplementary Files

Supplementary Table S1. Mouse Strains

**Strain**	**Gender**	**Vendor**[a]
C57BL/6	Female	Taconic
RAG2-/-	Female	Taconic
C57BL/6	Female	Jackson Laboratory
MyD88-/-	Female	Jackson Laboratory
TLR2-/-	Female	Jackson Laboratory
TLR4-/-	Female	Jackson Laboratory
FVB/N	Male/Female	Charles River Laboratories
Le^b transgenic	Male/Female	Colony maintained

[a] Jackson Laboratory, Sacramento, CA, USA; Taconic, Germantown, NY, USA; Charles River Laboratories, Wilmington, MA, USA

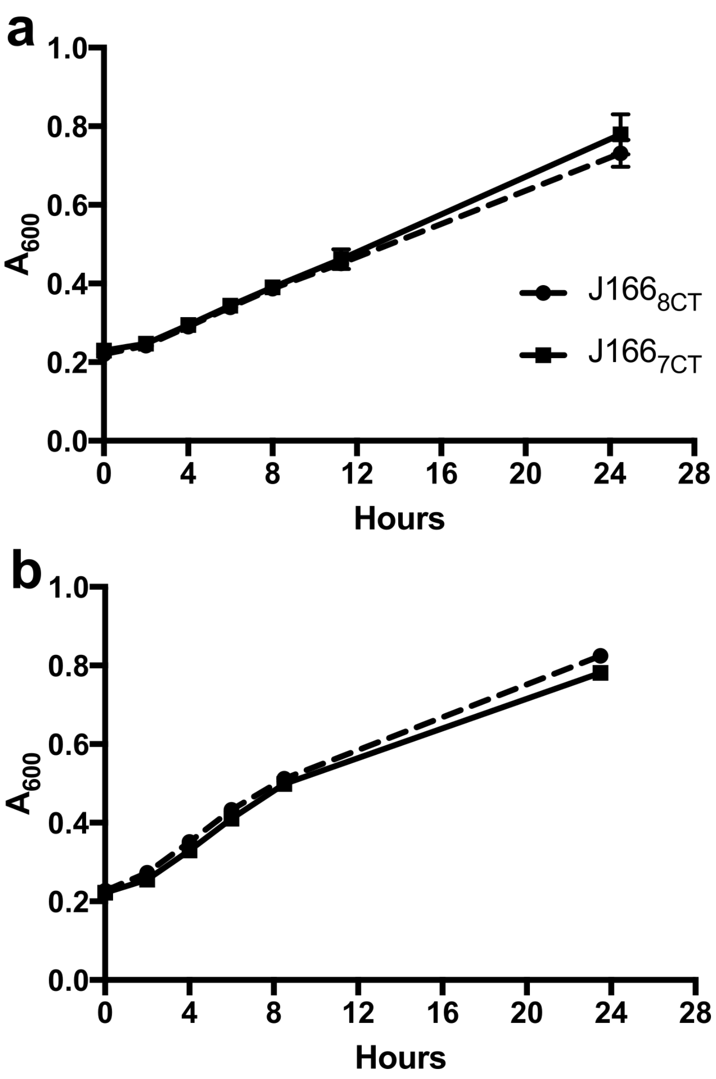

Supplementary Figure S1. Growth curves of J166$_{8CT}$ and J166$_{7CT}$. Mean (±SEM) A_{600} for 3 individual clones of J166$_{8CT}$ and J166$_{7CT}$ grown in Brucella broth overnight showed no differences in growth rate. The experiment was conducted twice (A and B) to ensure that subtle, non-significant variation in growth rate was not robust.

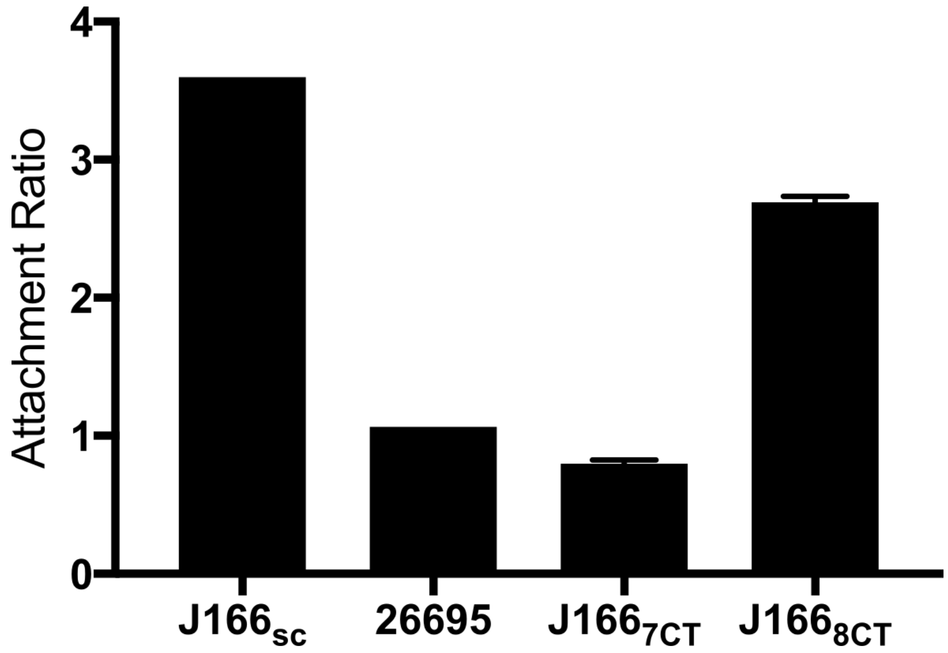

Supplementary Figure S2. Le^b ELISA assay to detect functional BabA expression in *H. pylori* clones. Clones of J166sc engineered with 8 or 7 CT repeats in the 5' end of *babA* were evaluated for attachment to Le^b using an ELISA. The average attachment ratios for three clones of $J166_{8CT}$ and $J166_{7CT}$ are shown. *H. pylori* $J166_{SC}$ and 26695 served as positive and negative controls, respectively.

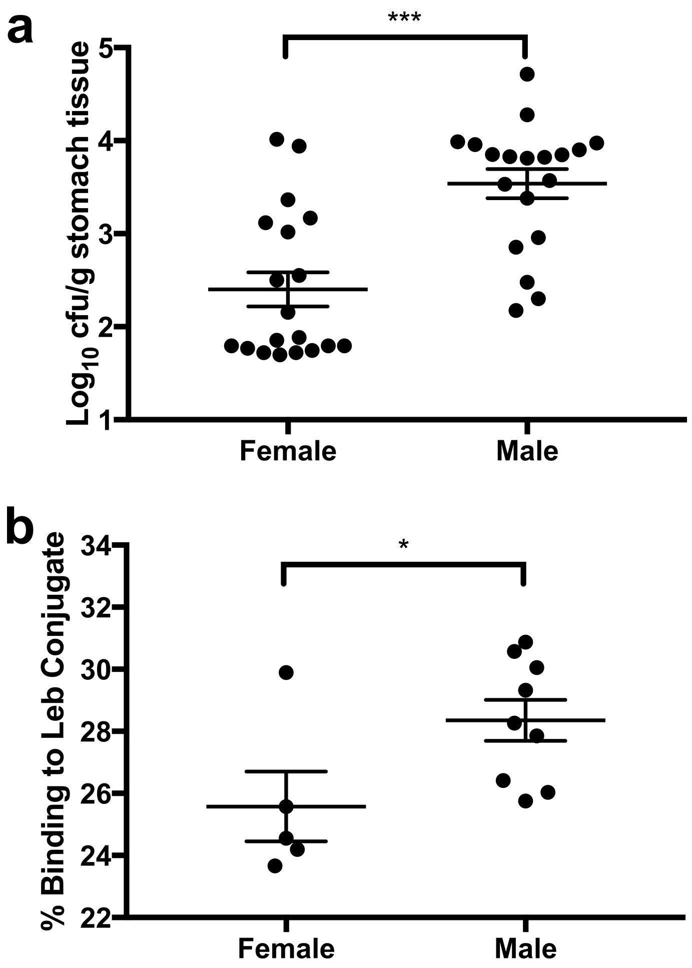

Supplementary Figure S3. *H. pylori* J99 colonization and expression of BabA is greater in male than female Le^{b} mice. Experiments were approved by the local ethical committee for animal studies at the Medical Faculty of Umeå University, Umeå, Sweden (A 120-06 from 10.11.2006). *H. pylori* J99[1,2] was grown on Brucella agar for 24 hours and suspended in Brucella broth at 10^8 CFU/mL. Mice were challenged intragastrically with 200μL (2 x 10^7 CFU) of bacterial suspension twice per week for two weeks using a ball-tipped disposable feeding needle. Four weeks after the last dose was administered, the homogenized stomach tissues were cultured on Brucella agar. A) *H. pylori* colonization of each mouse stomach, as determined by enumeration of *H. pylori* colonies when homogenized stomach tissues were plated on Brucella agar, is shown as CFU/gram of stomach tissue. Ten female mice that had no detectable colonization are shown at the limit of detection. B) Relative expression of functional BabA (binding to Le^{b}) was determined using the RIA assay to analyze total cultured *H. pylori* stomach contents from a subset of the mice. ***P = 0.0002, *P<0.05 (Mann-Whitney U)

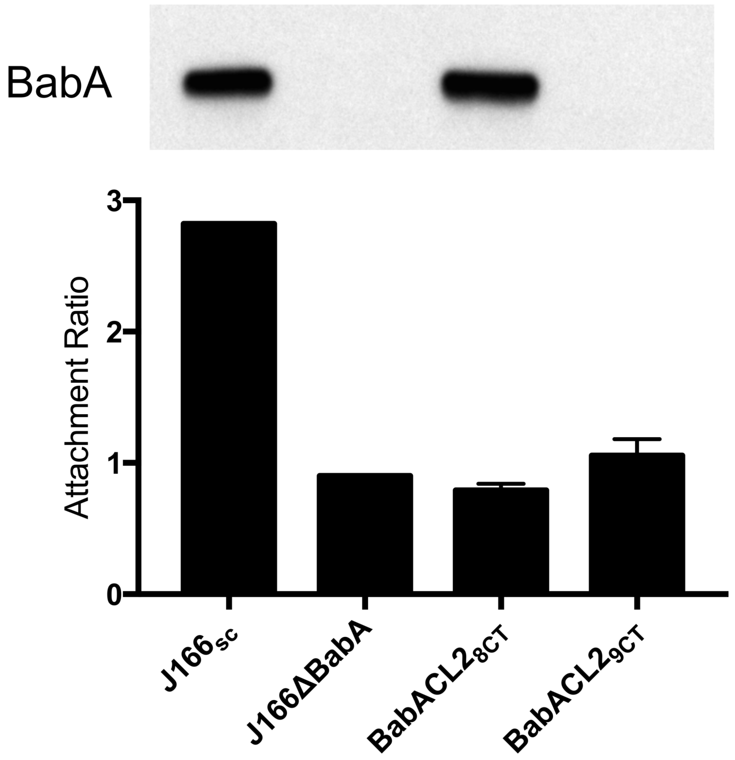

Supplementary Figure S4. Anti-BabA western blot and Le^b ELISA assay to detect BabA expression and function in *H. pylori* clones. Clones of BabACL2 with 8 or 9 CT repeats in the 5' end of *babA* were evaluated for expression of BabA by western blot (upper panel) and attachment to Le^b using an ELISA (lower panel). The average attachment ratios for three clones of BabACL2$_{8CT}$ and BabACL2$_{9CT}$ are shown. *H. pylori* J166$_{SC}$ and J166ΔBabA served as positive and negative controls, respectively.

Supplementary References

1. Alm, R. A. *et al.* Genomic-sequence comparison of two unrelated isolates of the human gastric pathogen Helicobacter pylori. *Nature* **397**, 176-180, doi:10.1038/16495 (1999).
2. Styer, C. M. *et al.* Expression of the BabA adhesin during experimental infection with Helicobacter pylori. *Infect Immun* **78**, 1593-1600, doi:10.1128/IAI.01297-09 (2010).
